# Constitutive activation of NF-κB signaling by NOTCH1 mutations in chronic lymphocytic leukemia

**DOI:** 10.3892/or.2015.3762

**Published:** 2015-01-29

**Authors:** ZHEN-SHU XU, JU-SHUN ZHANG, JING-YAN ZHANG, SHUN-QUAN WU, DONG-LIAN XIONG, HUI-JUN CHEN, ZHI-ZHE CHEN, RONG ZHAN

**Affiliations:** Fujian Institute of Hematology, Fujian Provincial Key Laboratory of Hematology, Fujian Medical University Union Hospital, Fuzhou, P.R. China

**Keywords:** chronic lymphocytic leukemia, NF-κB, NOTCH1

## Abstract

NOTCH1 mutations occur in approximately 10% of patients with chronic lymphocytic leukemia (CLL). However, the relationship between the genetic aberrations and tumor cell drug resistance or disease progression remains unclear. Frameshift deletions were detected by gene sequencing in the NOTCH1 PEST domain in three naive CLL patients. These mutations were associated with chromosomal abnormalities including trisomy 12 or 13q deletion. Of note, one of the patients developed Richter’s transformation during FCR treatment. Immunofluorescent and western blot analyses revealed a markedly higher intracellular domain of NOTCH (ICN) expression in the mutated cells compared with their unmutated counterparts and normal CD19^+^ B lymphocytes (P<0.01 and P<0.001, respectively). In addition, strong DNA-κB binding activities were observed in the mutant cells by gel shift assays. RT-PCR analysis revealed elevated RelA mRNA expression in the mutant cells, while RelB levels were variable. Reduced levels of RelA and RelB mRNA were observed in unmutated CLL and normal B cells. Compared to unmutated CLL and normal B cells, increased apoptosis occurred in the mutant cells in the presence of GSI (ICN inhibitor) and PDTC (NF-κB inhibitor), particularly under the synergistic effects of the two drugs (P=0.03). Moreover, IKKα and IKKβ, the active components in the NF-κB pathway, were markedly inhibited following prolonged treatment with GSI and PDTC. These results suggested that NOTCH1 mutations constitutively activate the NF-κB signaling pathway in CLL, which is likely related to ICN overexpression, indicating NOTCH1 and NF-κB as potential therapeutic targets in the treatment of CLL.

## Introduction

NF-κB is a family of proteins, including RelA, RelB, c-Rel, NF-κBl, and NF-κB2, which play a critical role in a variety of biological activities such as lymphocyte development, and lymphoid tumor cell proliferation and apoptosis (1). The function of the NF-κB signaling pathway in the proliferation and apoptosis of chronic lymphocytic leukemia (CLL) cells is a hotspot in cancer research and is therefore considered a promising therapeutic target for the treatment of CLL (2). Findings of recent studies have shown a frequency of only ~10% for NOTCH1 mutations in CLL at diagnosis, although this rate may reach 30% with the disease progressing towards Richter’s transformation or chemotherapy refractory CLL (3,4). It remains unclear whether NOTCH1 mutations affect NF-κB signaling, subsequently leading to drug resistance and disease progression. Therefore, we assessed 3 patients with features of CLL carrying NOTCH1 mutations, and investigated in detail the NOTCH1 mutation-induced constitutive activation of NF-κB signaling.

## Materials and methods

### Cells

The primary CLL cells were derived from samples obtained from naive CLL patients diagnosed at Union hospital of Fujian Medical University (Fuzhou, China). Normal bone marrow controls were collected from healthy donors. Written informed consent was provided by each participant. The study was approved by the institutional review board and conducted in accordance with the Declaration of Helsinki.

### Reagents and devices

Anti-human-CD19, CD5 and CD20 antibodies were purchased from BD Biosciences (San Diego, CA, USA). The human CD19^+^ magnetic bead sorting kit was obtained from Miltenyi Biotec (Shanghai, China). GSI and PDTC were purchased from Sigma-Aldrich Corp. (Shanghai, China). Flow cytometry kits were provided by BD Biosciences, and the ABI PCR instrument was purchased from ABI Biosystems (Shanghai, China).

### Isolation of CLL- and normal CD19^+^ B cells

Mononuclear cells (MNCs) were isolated from 5 ml of anti-coagulated bone marrow samples from CLL patients by density gradient centrifugation using the lymphocyte separation medium. Subsequently, CD19^+^ cells were sorted using CD19^+^ magnetic beads and stained with anti-CD19, CD5 and CD20 antibodies. Flow cytometric analysis showed a purity of ≥95% for CD5^+^CD19^+^ cells. As control samples, CD19^+^ cells from 10 ml of bone marrow samples from 5 healthy individuals were isolated with a final purity of ≥90%.

### Chromosomal karyotype analysis

Bone marrow cells were briefly cultured and processed according to conventional R-banding techniques. The probes used in FISH included the centromeric (CSP12), 11q22-23 deletion (ATM), 17p13 deletion (P53), and 13q14 deletion (RB1 and D13S25) probes obtained from Abbott (Beijing, China).

### Gene sequencing

Gene mutation analysis was performed by amplification of the IgVH and NOTCH1 fragments by RT-PCR. After purification, the PCR products underwent direct sequencing. The sequencing results were analyzed with the V-QUEST software, and IgVH mutations were validated with >2% base alteration as compared to the germline gene.

### Immunophenotyping and detection of Zap70 and CD38 by flow cytometry

Cells (1×10^6^) were incubated with the corresponding antibodies for 5 min at room temperature after washing with ice-cold FACS buffer. The cells were then resuspended to obtain single-cell suspensions after routine washes and examined by flow cytometry (BD Biosciences). The cells were designated as Zap70^+^ and CD38^+^ with >20% cells staining positive for these markers, respectively.

### Immunofluorescence analysis of ICN expression

After fixation with 4% formaldehyde and blocking (3% BSA-PBS), the cells were incubated with primary antibodies and fluorescence-conjugated secondary antibodies. After DAPI nuclear counterstaining, the cells were observed by fluorescence microscopy.

### Western blot detection of ICN expression

The cells were collected, lysed and boiled for 8 min in loading buffer. The resulting protein samples were subjected to polyacrylamide gel electrophoresis followed by membrane transfer. The membrane was sequentially incubated with anti-ICN primary- and horseradish peroxidase-conjugated secondary antibodies (eBioscience) prior to development.

### RT-PCR

One-step TRIzol extraction of total RNA was performed. cDNA was synthesized using primers designed with the Blast-Primer software. The nucleotide sequences of the forward primers of RelA and RelB were 5′-GACCGCT GCATCCACAGTTT-3′ and 5′-CCCCTACAATGCTGGCTCC CTGAA-3′ respectively. RT-PCR was conducted on an ABI PCR instrument using β-actin as an internal control.

### Measurement of NF-κB activity

The nuclear proteins were isolated and the DNA binding activity of NF-κB was measured using a TransAM™ ELISA detection kit (Shenzhen, China) according to the manufacturer’s instructions. Briefly, nuclear proteins were incubated with a biotin-labeled κB oligonucleo-tide probe to form a binding complex, which was then isolated by non-denaturing acrylamide gel shift, followed by infrared imaging to assess NF-κB activity.

### Assessment of apoptosis by flow cytometry

Cells (1×10^6^) were collected at different time points after drug treatment and incubated with Annexin V in combination with other antibodies for 5 min at room temperature. After washing, the cells were resuspended to obtain single-cell suspensions, which were analyzed via flow cytometry. The apoptotic rate was calculated as: (drug-induced apoptotic cell number - spontaneous apoptotic cell number) ×100/(100 − control group).

### Statistical analysis

The experiments were repeated for a total of three times. The Student’s t-test was used to compare the means of two independent samples with the SPSS16.0 software (SPSS Inc., Armonk, NY, USA). P<0.05 was considered statistically significant.

## Results

The gene sequencing results for samples from the 3 naive CLL patients are shown in Table I. All patients presented a CT frameshift deletion in the PEST domain of the *NOTCH1* gene, which was associated with an abnormal chromosomal karyotype such as trisomy 12 and 13q deletion. The patients carrying NOTCH1 mutations had a poor prognosis. One of the patients experienced disease progression characterized by fever and lymph node enlargement during the 4th course of FCR therapy. Lymph node biopsy suggested diffuse large B-cell lymphoma transformation.

**Figure 1:**
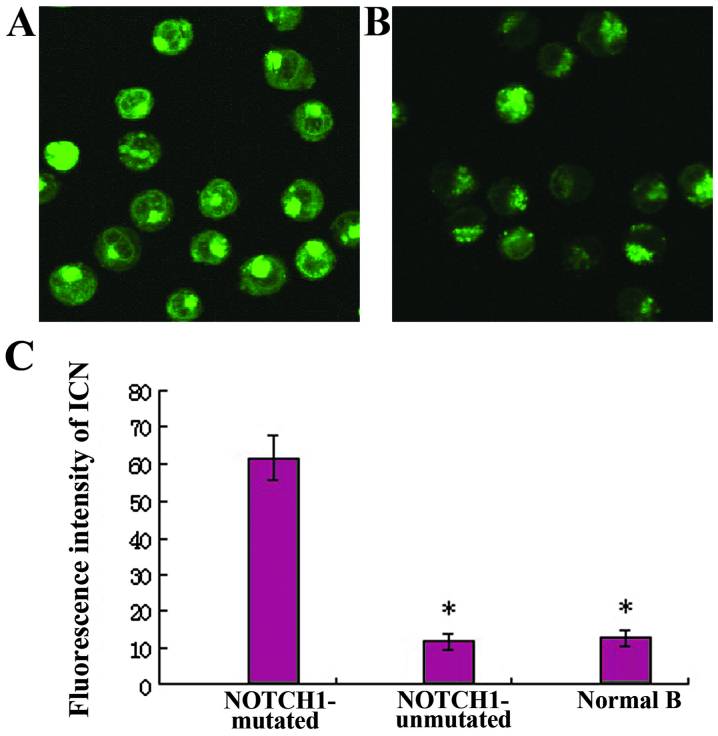
High expression of ICN proteins in the cytoplasm and nuclei of NOTCH1-mutated CLL cells, detected by immunofluorescence (x400). The images are representative of three (A) mutated and (B) five unmutated CLL cells. (C) In CLL cells carrying NOTCH1 mutations, the ICN protein was abundantly expressed in the cytoplasm and nuclei, at significantly higher levels than that observed in unmutated and normal CD19^+^ B cells (^*^P<0.01).

**Figure 2:**
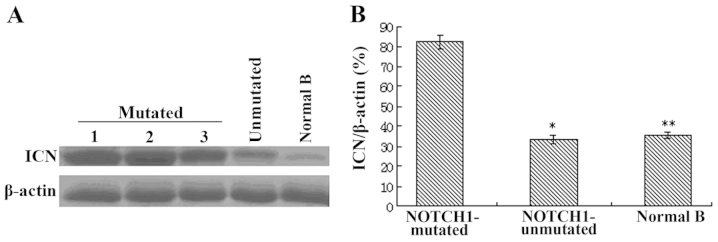
(A) High expression of ICN proteins in NOTCH1-mutated CLL cells by western blot assay. Western blot analysis showing overexpression of the ICN proteins in NOTCH1-mutated CLL cells from the three patients, whereas ICN levels were significantly reduced in (B) unmutated and normal B cells (^*^P<0.01 and ^**^P<0.001). Data are representative of three independent experiments.

In CLL cells carrying NOTCH1 mutations, the ICN protein was abundantly expressed in the cytoplasm and nuclei, at significantly higher levels than that observed in unmutated and normal CD19^+^ B cells by immunofluorescence (P<0.01) (Fig. 1). Western blot analysis revealed similar results, with markedly reduced ICN protein levels in unmutated- and normal B cells compared with NOTCH1-mutated CLL cells (P<0.01 and P<0.001) (Fig. 2).

**Figure 3:**
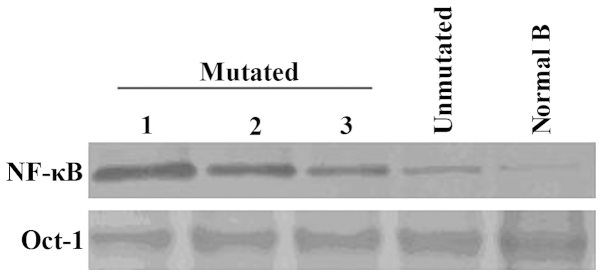
Comparison of NF-κB DNA-binding activities in three NOTCH1-mutated CLL, one unmutated CLL and one normal B-cell case by gel mobility shift assay.

NF-κB activity assessment showed robust DNA-binding activity of the NF-κB nuclear protein in NOTCH1-mutated CLL cells, whereas only weak binding activity was detected in unmutated- and normal B lymphocytes (Fig. 3). In addition, ELISA showed markedly increased RelA and RelB activities in mutated CLL cells (0.56±0.03 and 0.28±0.05, respectively) compared with unmutated CLL cells (0.19±0.01 and 0.08±0.00, respectively, P<0.01) and normal B-cells (0.01±0.0 and 0.007±0.00, respectively, P<0.01).

**Figure 4:**
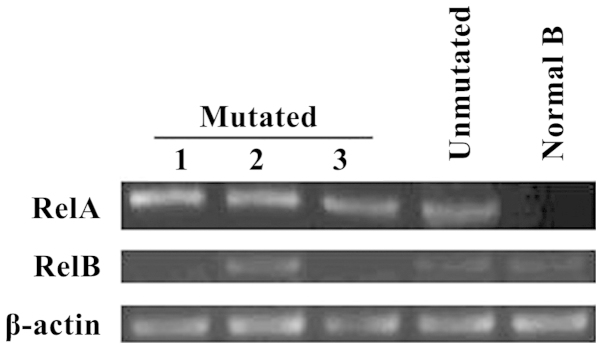
mRNA expression of RelA and RelB in three NOTCH1-mutated CLL, one unmutated CLL and one normal B-cell cases, assessed by RT-PCR.

The mRNA levels of RelA and RelB in CLL cells were examined by RT-PCR. As shown in Fig. 4, the expression of RelA and RelB mRNA in CLL cells varied considerably. Regardless of the presence or absence of NOTCH1 mutations, RelA mRNA was always detected in CLL cells. However, RelB mRNA was only found in the second patient carrying NOTCH1 mutations.

**Figure 5:**
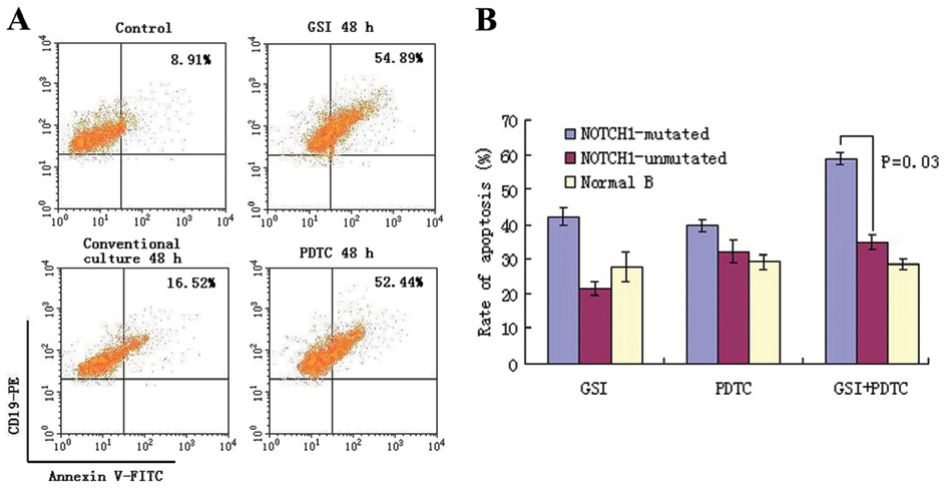
(A) Representative FACS analysis showing significantly increased apoptosis of CLL cells at 48 h after GSI and PDTC treatments, respectively (n=3). (B) The ratio of apoptotic cells was significantly elevated following synergistic effects of the two drugs, and was much higher than that in the unmutated group (P=0.03).

**Figure 6:**
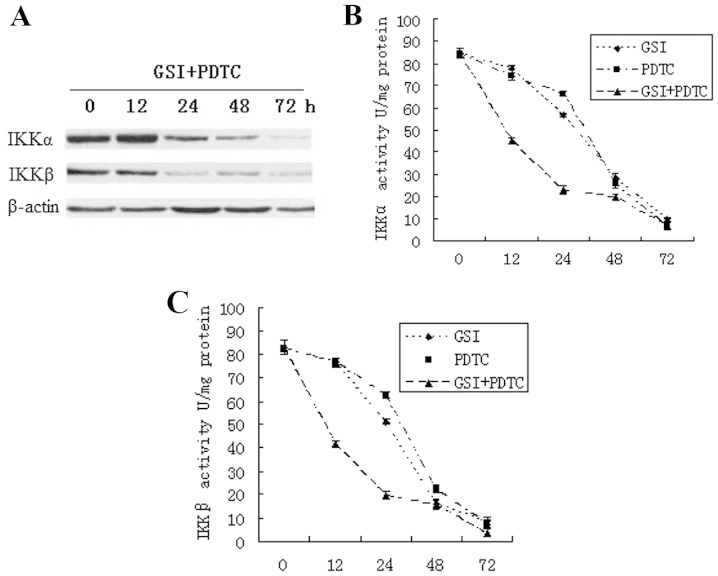
(A) Western blot analysis revealed obvious inhibition of (B) IKKα and (C) IKKβ, the active proteins in the NF-κB pathway, following prolonged treatment of GSI and PDTC.

We hypothesized that NF-κB inhibition was able to induce apoptosis in CLL cells. As shown in Fig. 5, the ratios of apoptotic cells in NOTCH1-mutated CLL cells were significantly increased following treatment with GSI (ICN-specific inhibitor) and PDTC (NF-κB-specific inhibitor) compared with those obtained in unmutated- and normal B cells, and this effect was even greater when the two drugs were used (P=0.03). Western blotting confirmed suppression of the active IKKα and IKKβ together with the prolonged treatment of GSI in combination with PDTC (Fig. 6).

## Discussion

Results of the present study are in agreement with those of previous reports, which indicated that the clinical factors, biological characteristics, treatment response and prognosis of CLL patients in China vary from those of other countries (5,6). The median survival time of CLL patients was ~67 months, with many individuals developing drug resistance or progressing to aggressive lymphoma, or Richter’s syndrome, during the therapeutic procedure. In this case, the patients showed poorer prognosis with survival for only 5–8 months. Thus, it is important to further assess the mechanisms involved in the malignant transformation of CLL and explore novel therapeutic targets for its treatment.

The NF-κB signaling pathway has been shown to play a critical role in the promotion of proliferation and anti-apoptosis of B-lymphoid tumor cells (1,7,8). Unlike other lymphoid tumors, IKK kinase activation is indispensable to the NF-κB signaling pathway in CLL cells (9). Ubiquitination by the CYLD enzyme K63, the adapter protein of IKK kinase, can effectively regulate NF-κB signaling. Other regulatory factors such as NOTCH1 and Bcl-10 also have crucial functions in this pathway. For instance, through direct or indirect effects on CYLD ubiquitination, NOTCH1 constitutively activates NF-κB signaling in CLL cells, which results in resistance to apoptosis and consequently contributes to the development and progression of CLL (10,11).

Recent studies have also shown that mutations of NOTCH1 promoted the release and stabilization of ICN, which in turn excessively inhibited CLL cell apoptosis and to a greater extent, led to aggressive progression to Richter’s syndrome (12,13). This may be a consequence of the direct activation of NF-κB signaling by ICN following NOTCH1 mutation (3). Although no exact mechanisms were revealed for ICN activation of NF-κB, these findings demonstrated that ICN, a dominant active form of NOTCH1, play an indispensable role in the NF-κB signaling pathway, particularly following NOTCH1 mutation. Therefore, NOTCH1 mutation is considered a critical indicator for unfavorable prognosis in CLL (14–16).

Nevertheless, NOTCH1 mutation is not a common event in CLL. Shen *et al* (17), Giulia *et al* (3), and Shedden *et al* (4) performed second-generation sequencing and found an incidence of ~10% for NOTCH1 mutations in CLL patients at diagnosis. However, this incidence may reach 30% following aggressive transformation of the disease or occurrence of chemotherapy resistance, particularly in association with cytogenetic abnormalities including 17p- and trisomy 12 (18). In this case, poor prognosis is expected for patients. It is conceivable that certain connections exist between NOTCH1 mutations, NF-κB signal aberrations and malignant transformation of the disease. In this study, all three CLL patients had additional abnormal karyotype of trisomy 12 or 13q-deletion, together with unfavorable prognosis: one of these patients rapidly progressed to disease transformation into diffuse large B-cell lymphoma (Richter’s syndrome).

Further sequencing analysis revealed dinucleotide frame-shift deletion in the PEST domain of NOTCH1. Previously, such mutations identified in NOTCH1 exon 34 resulted in truncated C-terminal PEST domain. Consequently, ICN, the protein product of the truncated PEST domain mediated the sustained activation of the NF-κB signaling pathway (4). Additional studies have indicated enriched ICN proteins in the cytoplasm and nuclei of mutated CLL cells exhibiting strong NF-κB-dependent anti-apoptotic properties. This may constitute a critical molecular basis for the aggressive transformation of CLL cells. An important issue is how ICN proteins affect NF-κB signaling. Different findings have been provided from investigations on assorted lymphoid tumors. For instance, Schwarzer *et al* found that ICN directly activates IKK kinase in Hodgkin lymphoma cells, thereby activating the NF-κB signaling pathway (19).

D’Altri *et al* demonstrated that ICN constitutively activates NF-κB through CYLD inhibition and NF-κB de-ubiquitination in T-ALL cells (20). Following treatment of NOTCH1-mutated cells with specific inhibitors, we observed a marked inhibition of IKKα and IKKβ, accompanied by prolonged GSI and PDTC effects. These results suggest that NOTCH1 mutations in CLL lead to constitutive activation of NF-κB signaling, likely due to ICN overexpression. Nevertheless, it remains to be elucidated how ICN activates the NF-κB signaling pathway following NOTCH1 mutation, which is possibly a key mechanism involved in the malignant transformation of CLL, and requires further investigation.

## Figures and Tables

**Table I:** Clinical factors and therapeutic outcomes of the three naive CLL patients.

Characteristics	Patient 1	Patient 2	Patient 3
Age (years)	56	76	61
Gender	F	M	M
ECOG	0	1	2
Binet stage	B	C	B
β2-MG	3.5 mg/l	3.8 mg/l	3.2 mg/l
Zap-70	+	+	−
CD38	−	−	−
Karyotype	47,XX,+12[19] /46XX[1]	46,XY,del(13)(q14q21) [8]/46,XY[12]	46,XY,+12,del(13) (q13q21)[5]/46,XY[15]
IgVH	Mutated	Unmutated	Mutated
NOTCH1 nucleotide	c7541_7542het_delCT	c7541_7542het_delCT	c7541_7542het_delCT
Treatment and outcome	FCR 4 courses Richter transformation	FCR 4 courses + R-CHOP 2 courses PR	FCR 3 courses died due to cerebral hemorrhage

CLL, chronic lymphocytic leukemia; IgVH unmutated, ≥98% homology with germline; F, FCR-fludarabine; C, cyclophosphamide, R, rituximab; PR, partial remission.
